# The 4^th^ edition of the Romanian National Neurology Forum: NNF policies in times of uncertainty: between pragmatism, progress, and partnerships

**DOI:** 10.25122/jml-2026-1008

**Published:** 2026-05

**Authors:** Alexandra Gherman, Stefana-Andrada Dobran, Dafin Fior Muresanu

**Affiliations:** 1 RoNeuro Institute for Neurological Research and Diagnostic, Cluj-Napoca, Romania; 2 Department of Neuroscience, Iuliu Hatieganu University of Medicine and Pharmacy, Cluj-Napoca, Romania

## The 4^th^ National Neurology Forum: NNF policies in times of uncertainty: between pragmatism, progress, and partnerships

Held on April 23-24 in Bucharest, at the Palace of the Patriarchate, the 2026 edition of the Romanian National Neurology Forum (NNF) marked a paradigm shift in the transition of Romanian neurology from the strategic to the implementation phase, focusing on generating effective solutions dedicated to patients and optimising the health system through increased collaboration among patients, medical specialists, authorities, and industry representatives. The NNF continuously advances and emerges as a unique platform for debate and cooperation on strategies and public health policies – laying the foundation for the Annual Congress of the Romanian Neurology Society (June 3-5, 2026, Sinaia, Romania).

**Figure 1:**
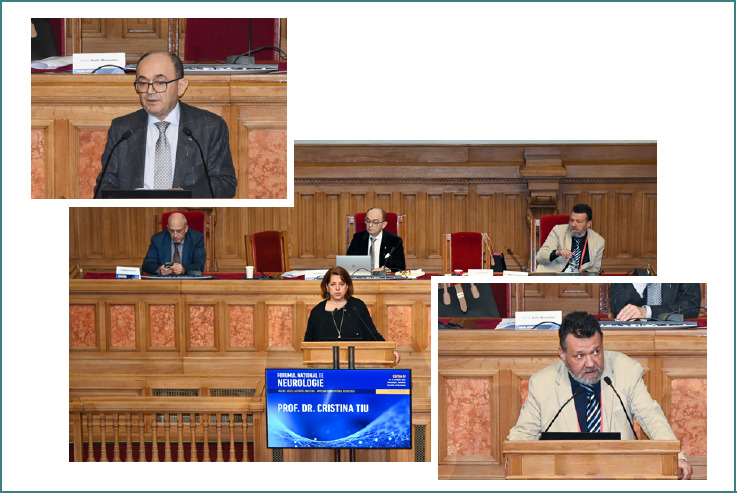
The opening of the National Neurology Forum with Prof. Dr Dafin Mureșanu, president-elect of the Romanian Society of Neurology (RSN) and president of the European Federation of Neurorehabilitation Societies (EFNR), Prof. Dr Cristina Tiu, Former President of the Romanian Society of Neurology, and Prof. Dr Bogdan O. Popescu, Vice-Rector of the Carol Davila University of Medicine and Pharmacy in Bucharest, joined by Prof. Dr Dragoș Vinereanu, Head of the Cardiology and Cardiovascular Surgery Department at the University Emergency Hospital Bucharest and Co-coordinator of the National Strategy for Combating Cardiovascular and Cerebrovascular Diseases

The 4^th^ edition of the Forum was organised and coordinated by Prof. Dr Fior - Dafin Muresanu, president-elect of the Romanian Society of Neurology (RSN) and president of the European Federation of Neurorehabilitation Societies (EFNR), Associate Prof. Cristina Panea – president of the Romanian Society of Neurology, alongside Prof. Dr Cristina Tiu, former president of the RSN, and Prof. Dr Bogdan O. Popescu, vice-rector of Carol Davila University of Medicine and Pharmacy (Figure 1), with the support of the Foundation of the Society for the Study of Neuroprotection and Neuroplasticity, Ministry of Health, Romanian Society of Neurology, Carol Davila University of Medicine and Pharmacy, Iuliu Hatieganu University of Medicine and Pharmacy, George Emil Palade University of Medicine, Pharmacy, Science and Technology of Targu Mures, and endorsed by the World Federation for Neurorehabilitation, the European Federation of Neurorehabilitation Societies, the European Stroke Organisation and the Academy for Multidisciplinary Neurotraumatology.

## THE Rare Neuro Alliance Romania

**Figure 2:**
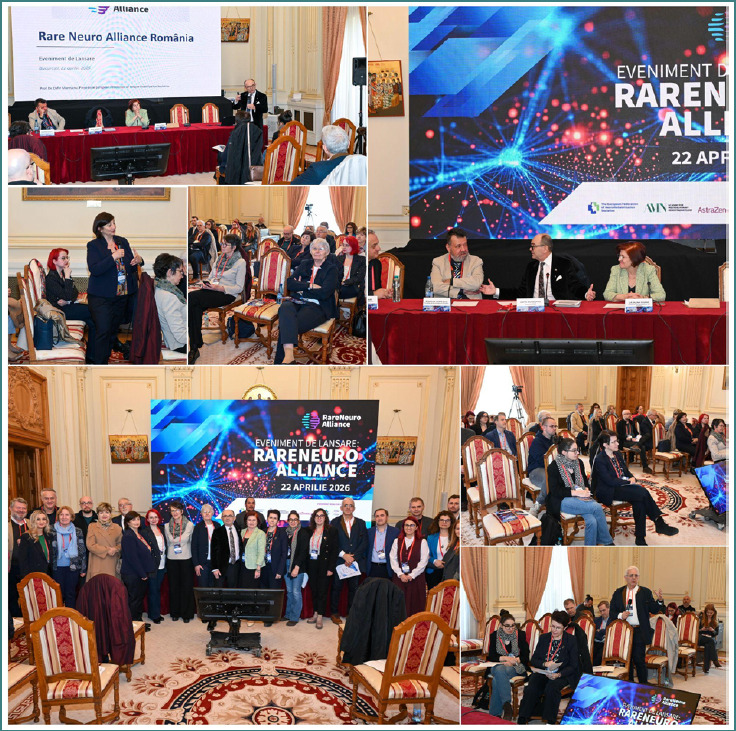
The Rare Neuro Alliance Romania press conference

On April 22^nd^, as a preamble to the National Neurology Forum, the **Rare Neuro Alliance Romania** was launched (Figure 2), in connection with other initiatives promoted by the European Health Council, aligned with European standards and reflecting the European vision on developing a specific framework dedicated to rare neurological diseases, to improve patient care and reduce the inequities in access to medical services.

“The Rare Neuro Alliance Romania initiative represents an essential step towards building a more equitable and efficient healthcare system for patients with rare neurological diseases. We aim to transform the way these patients are identified, diagnosed and treated, through real collaboration between specialists, institutions and patient organisations in a context where alignment with European initiatives, such as those promoted by the European Brain Council, provides us with the necessary framework to develop modern, integrated and sustainable solutions.”

*- Prof. Dr Fior-Dafin Muresanu*,

*president of the European Federation of Neurorehabilitation Societies*

Rare Neuro Alliance Romania will actively advocate for the acknowledgement of rare neurological diseases as a public health priority, the increase in awareness level for early diagnosis, and the implementation of an integrated model of patient care. Equally, this initiative is focused on developing a national reference network and capitalising on digitalisation and medical data to support efficient and resilient health policies.

## Insights from NNF

**Figure 3:**
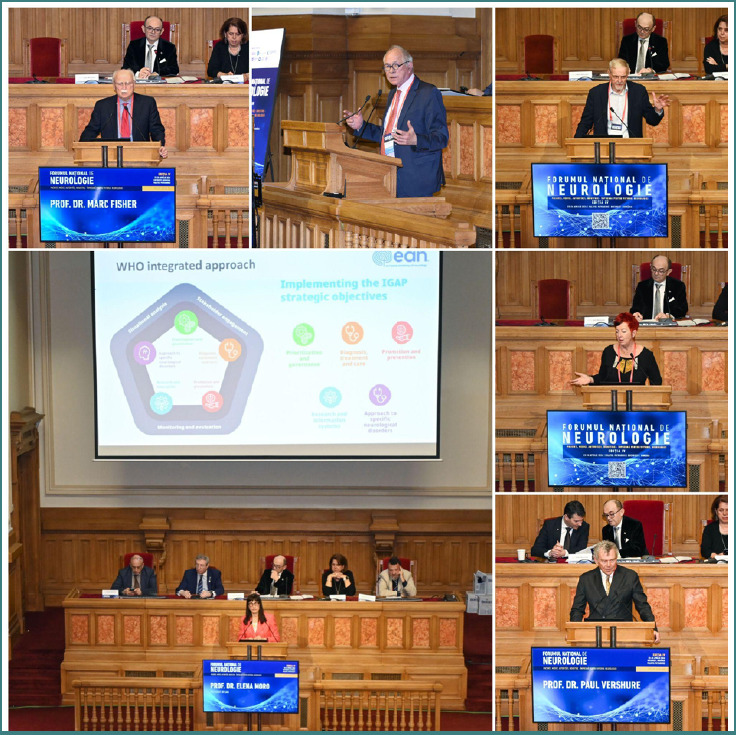
The Brain Health Mission Session | first row: Prof. Dr Marc Fisher, Former President of the World Stroke Organization; Prof. Dr Volker Hömberg, President of the World Federation for Neurorehabilitation (WFNR); Prof. Dr Natan Bornstein, Chairman of the Israeli Stroke Society; second row (left): Prof. Dr Elena Moro, President of the European Academy of Neurology (EAN), second row (right): Dr Dana Boering, Secretary General of the European Federation of Neurorehabilitation Societies (EFNR) & Prof. Dr Paul Vershure, Leader of the Synthetic Perceptive, Emotive and Cognitive Systems Laboratory, of the Institute for Bioengineering of Catalunya.

With more than 300 participants (online and on-site), the event featured a dynamic scientific program structured on joint sessions, interactive discussions and networking sessions (Annex 1). Thus, the ‘Brain Health Mission’ session (Figure 3) outlined the strategic cooperation with the European Academy of Neurology (EAN), and reunited world-renowned experts who contributed their international expertise to the Forum, among whom Prof. Dr Elena Moro – EAN president, Prof. Dr Mark Fisher – former president of the World Stroke Organisation, Prof. Dr Volker Hömberg, president of the World Federation of Neurorehabilitation, Prof. Dr Natan Bornstein, chairman of the Israeli Stroke Society, Dr Dana Boering, secretary general of the European Federation of Neurorehabilitation Societies, and Prof. Dr Paul Verschure, who leads the Synthetic Perceptive, Emotive and Cognitive Systems Laboratory, hosted by the Institute for Bioengineering of Catalunya.

Annex 1

## The core of NNF: Multidisciplinary Interactive Workshops

**Figure 4:**
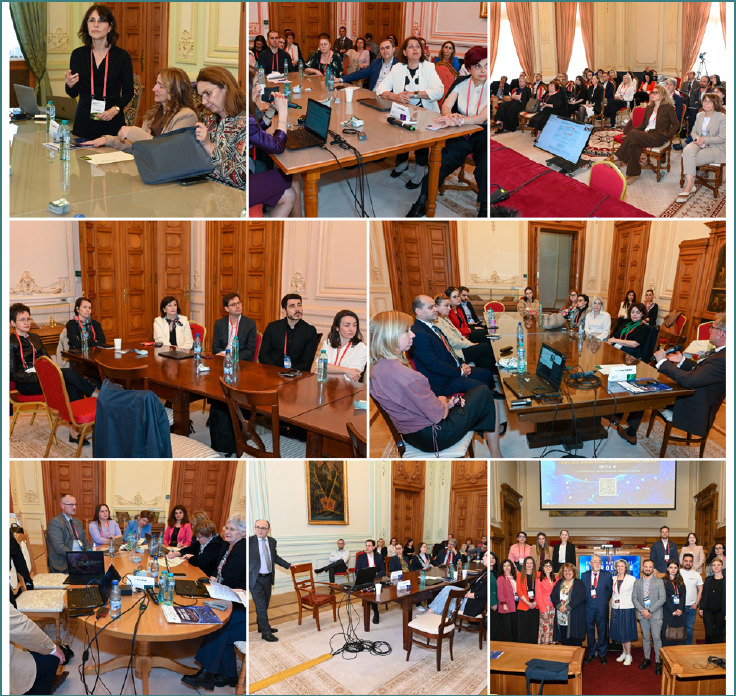
Workshops of the 4^th^ National Neurology Forum - first row: Epilepsy Workshop, Multiple Sclerosis Workshop, Neurodegenerative Diseases – Parkinson’s and Alzheimer’s Workshop; second row: Neuro-oncology Workshop and Neurotraumatology Workshop, third row: Rare Neurological Diseases in Romania Workshop, the Implementation of the National Cardiovascular and Cerebrovascular Disease Strategy Workshop and the Headache and Migraine Workshop.

Notably, compared to the 2025 edition which featured six distinct workshops, the 4^th^ edition offered participants the chance to engage in eight multidisciplinary workshops (Figure 4). New additions included the *Neuro-oncology* and *Neurotraumatology* workshops, alongside the already well-established and highly attended ones focused on the *Implementation of the National Cardiovascular and Cerebrovascular Disease Strategy, Neurodegenerative Diseases – Parkinson’s and Alzheimer’s, Multiple Sclerosis, Epilepsy, Headache and Migraine, and Rare Neurological Diseases in Romania*.

Thus, key aspects were addressed and debated during the discussions, with special attention placed on:

- The implementation of health policies, i.e., the paradigm shift from planning to action, in the framework brought forward the progress made within the National Strategy for Combating Cardiovascular and Cerebrovascular Diseases (SNBCC) 2025–2030, outlining the contribution of the academic environment, as well as the results obtained at the institutional level, also with the support of the National Health Insurance House;
- The interdisciplinary and multidisciplinary cooperation, e.g., neurology-cardiology partnerships in which the contribution of Prof. Dr Dragos Vinereanu, head of the Cardiology Unit within the Bucharest Emergency University Hospital, is essential to the development of an integrative approach in the management of complex pathologies and enhancing the dialogue between specialities to the benefit of the patient;
- The development of the Focus Group Neurotraumatology Romania as a strategic construct within the Academy for Multidisciplinary Traumatology under the coordination of Prof. Dr Dorel Sandesc, general manager of Pius Brinzeu County Emergency Clinical Hospital, Timisoara, who outlined that neurotrauma must be addressed as a major public health problem—with specific financing, a regionalized clinical pathway, centres of expertise, multidisciplinary teams active from the acute phase, and real continuity through recovery, social reintegration, and family and community support;
- The improvement of the quality of life for patients diagnosed with Multiple Sclerosis (MS) by means of specific measures, such as ease of access to treatment through open circuit pharmacies for those therapies that do not necessitate hospitalisation—a very useful tool that would ease the administrative burden for the patients and make the medical activity more efficient, with important savings for the health system;
- The management of Parkinson's disease, i.e., optimising the clinical guidelines, simplifying the patients’ care pathway, and making access to device-assisted therapies (DAT) easily available;
- The optimisation of neuro-oncology patient care at the national level by (1) identifying solutions for training/recognition of neuro-oncologists, neuro-imagists, neuro-pathologists, and (2) gaining access to immunological, genetic, and molecular diagnostic tests, as well as to advanced imaging techniques, minimally invasive neurosurgical techniques, modern therapies, and palliative care. Other important advances would be the improvement of medical practices in neuro-oncology, the establishment of regional multidisciplinary medical committees for this sub-especialty, and the expansion of the National Neuro-Oncology Subcommittee to other related specialities, to standardise diagnostic and treatment practices in accordance with European ones, like the European Association of Neuro-Oncology (EANO) or the European Society for Medical Oncology (ESMO).

## Conclusions

The Romanian National Neurology Forum is already established as a genuine strategic network for the development of both the national and international field of neurology, and it continues to actively engage in and support the professional interdisciplinary dialogue, aimed at transforming communications in real, specific measures, with a clear and immediate impact on patients, patient care and the national health system.

**Figure 5:**
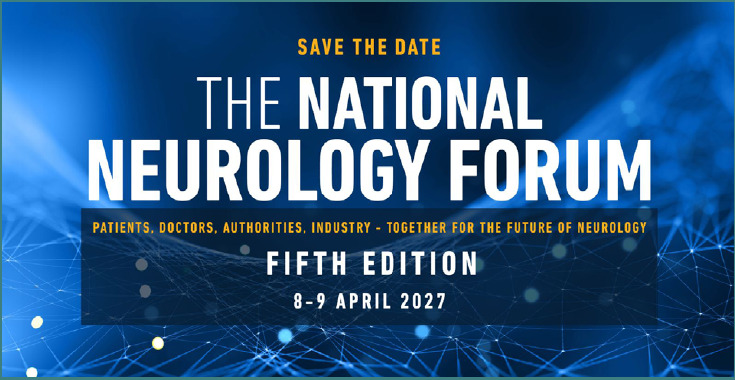
National Neurology Forum 2027

*Join us in 2027 and become one of our dialogue partners, and support us in advocating for the patients! (Figure 5)*

