# Supplementary material for: The 4th edition of the Romanian National Neurology Forum: NNF policies in times of uncertainty: between pragmatism, progress, and partnerships
Source: J Med Life. 2026 May;19(5):331–5. doi: 10.25122/jml-2026-1008 (PMC13389815; doi:10.25122/jml-2026-1008)
Supplement: Supplementary file 1 [file JMedLife-19-331-s001.pdf]

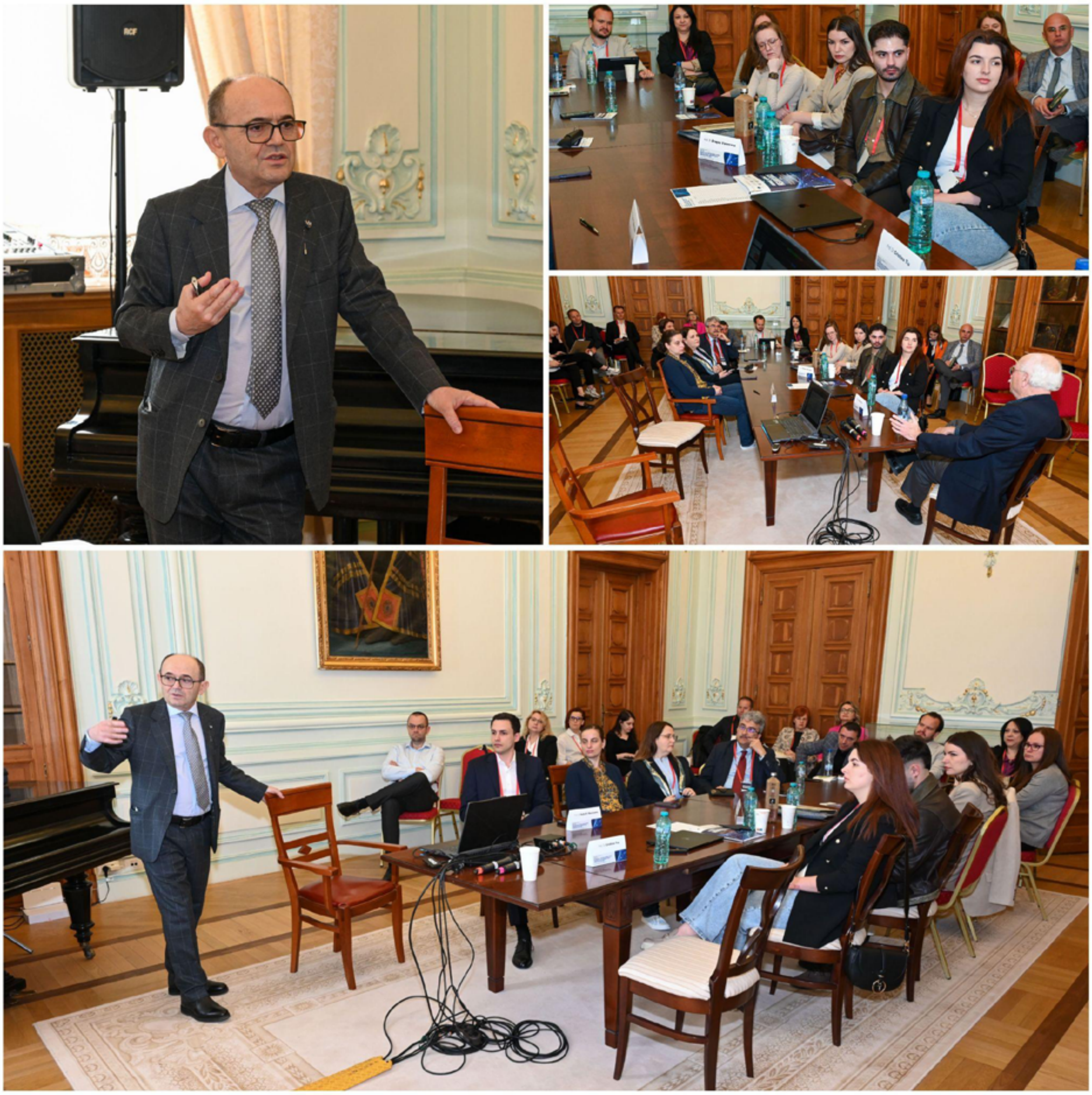

A. Photos from the Implementation of the National Cardiovascular and Cerebrovascular Disease Strategy Workshop coordinated by Prof. Dr Dafin Mureșanu, president-elect of the Romanian Society of Neurology (RSN) and president of the European Federation of Neurorehabilitation Societies (EFNR) & Prof. Dr Cristina Tiu, Former President of the Romanian Society of Neurology

© 2026 by the authors. This article is an open access article distributed under the terms and conditions of the Creative Commons Attribution (CC BY 4.0) license.

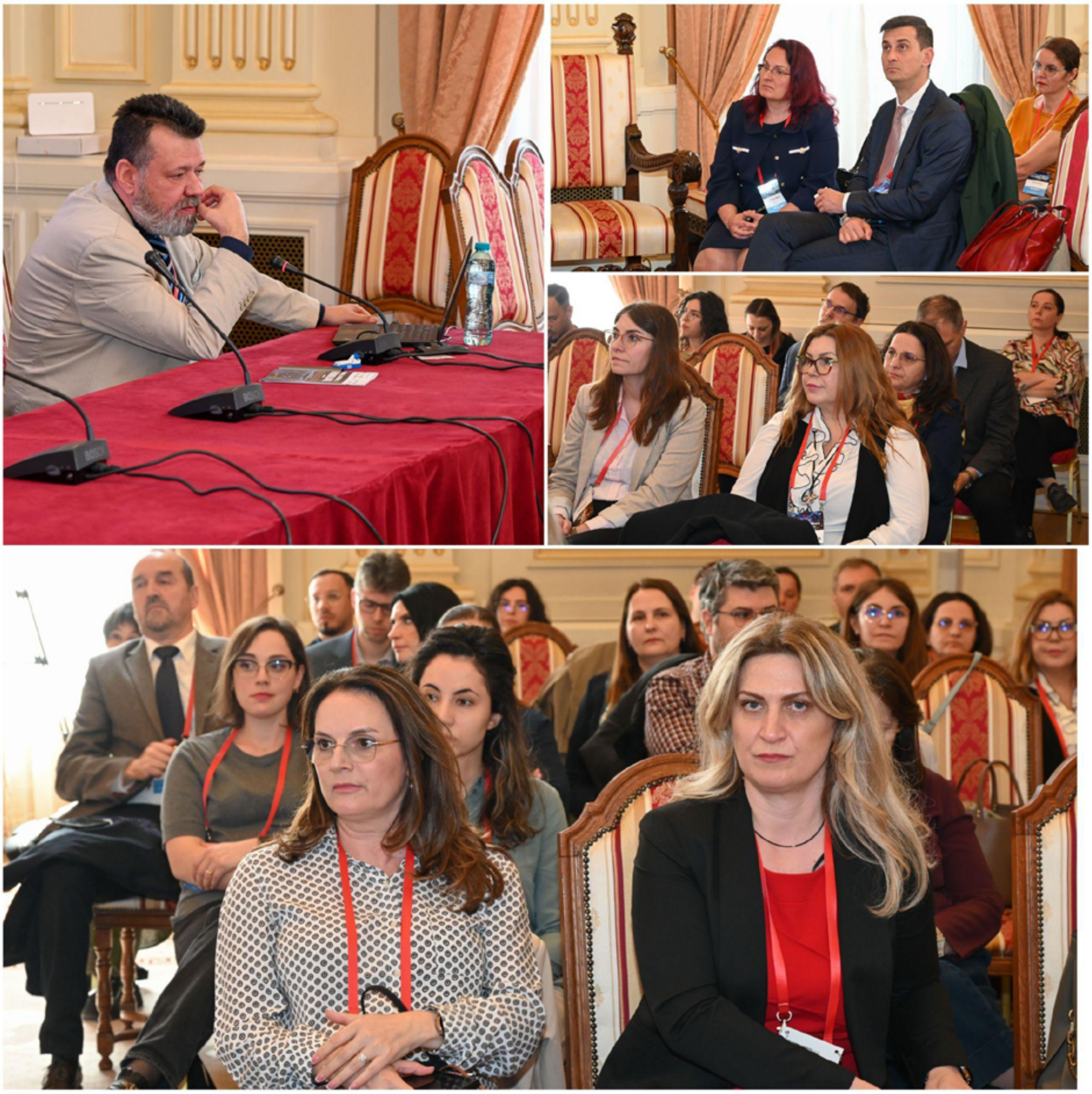

B. Photos from the Neurodegenerative Diseases – Parkinson's and Alzheimer's Workshop coordinated by Prof. Dr Bogdan Ovidiu Popescu, Vice-Rector of the Carol Davila University of Medicine and Pharmacy in Bucharest

© 2026 by the authors. This article is an open access article distributed under the terms and conditions of the Creative Commons Attribution (CC BY 4.0) license.

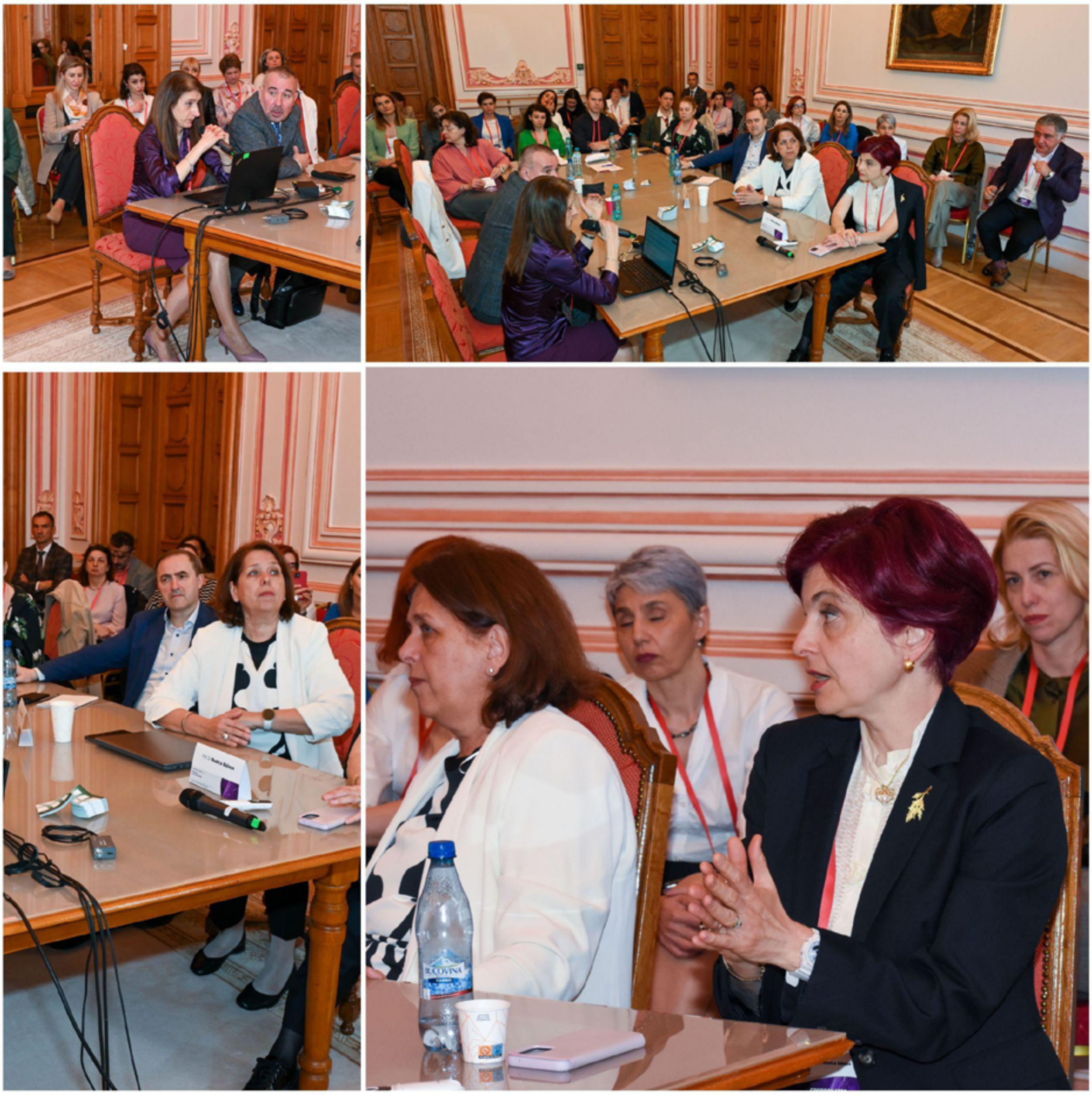

C. Photos from the Multiple Sclerosis Workshop, coordinated by Prof. Dr Cristina Tiu, former President of the Romanian Society of Neurology & Prof. Dr Rodica Bălașa, professor of neurology at the George Emil Palade University of Medicine, Pharmacy, Sciences and Technology of Targu Mures (UMFST)

© 2026 by the authors. This article is an open access article distributed under the terms and conditions of the Creative Commons Attribution (CC BY 4.0) license.

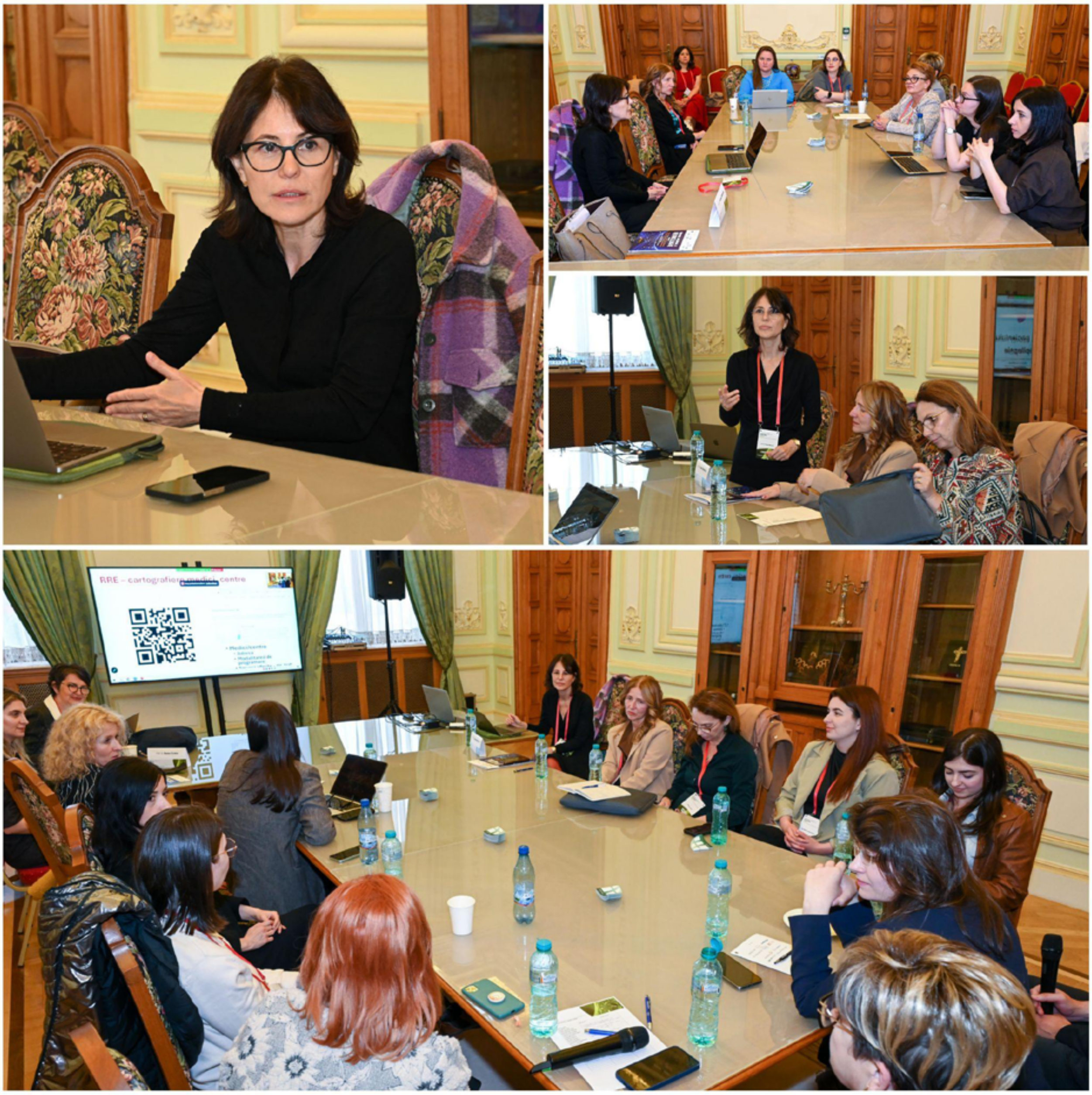

D. Photos from the Epilepsy Workshop, coordinated by Prof. Dr Dana Craiu, Head of the Department of Pediatric Neurology at the Faculty of Medicine, Carol Davila University of Medicine and Pharmacy in Bucharest & Assoc. Prof Dr Ioana Mîndruță, Associate Professor at the Department of Neurology, Carol Davila University of Medicine and Pharmacy, Bucharest and Senior Consultant Neurologist at the Epilepsy and Sleep Monitoring Unit, Clinical Neurophysiology Laboratory, Bucharest University Emergency Hospital

© 2026 by the authors. This article is an open access article distributed under the terms and conditions of the Creative Commons Attribution (CC BY 4.0) license.

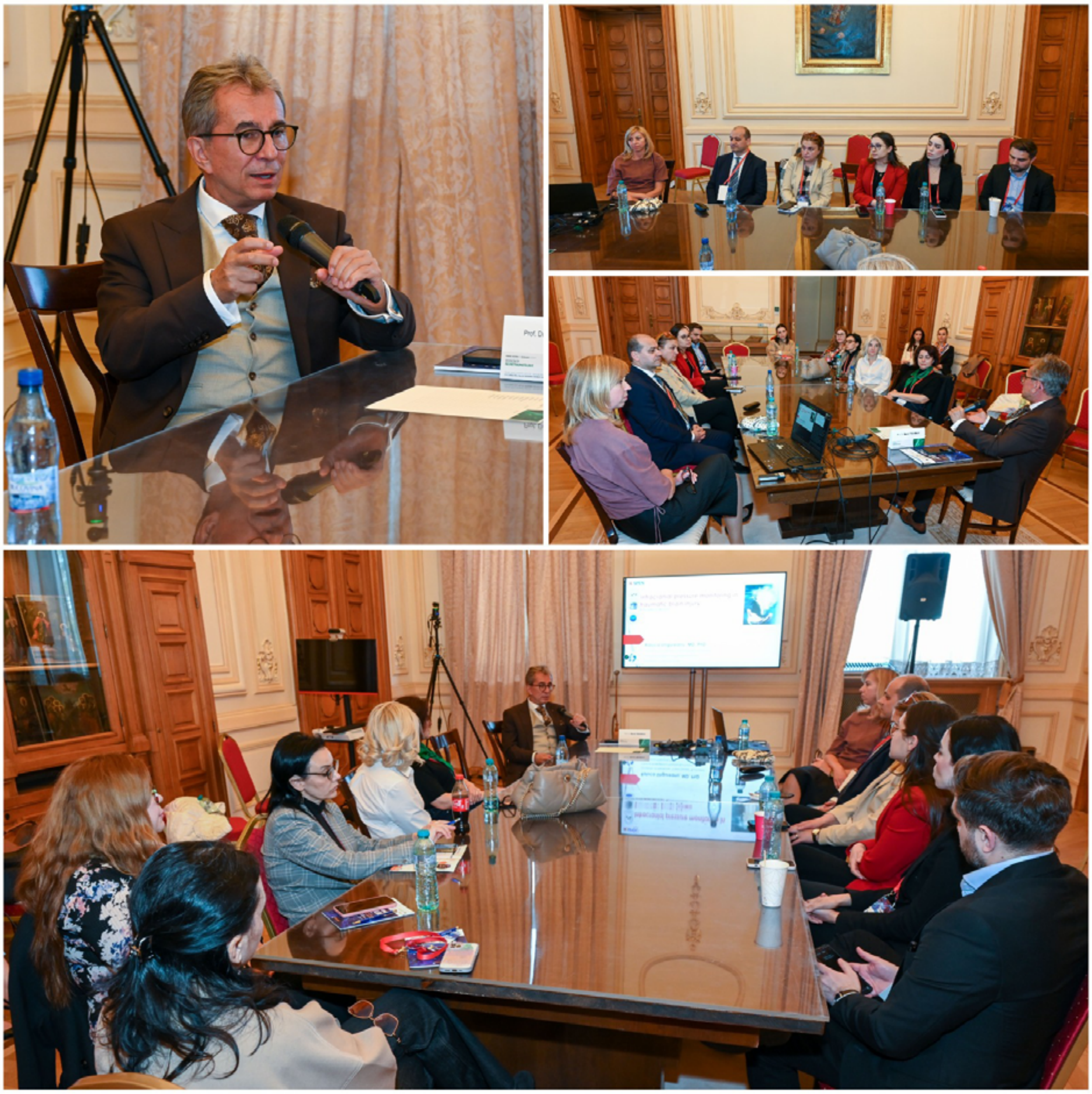

E. Photos from the Neurotraumatology Workshop, coordinated by Prof. Dr Dorel Săndesc, General Manager of Pius Brinzeu County Emergency Clinical Hospital, Timisoara

© 2026 by the authors. This article is an open access article distributed under the terms and conditions of the Creative Commons Attribution (CC BY 4.0) license.

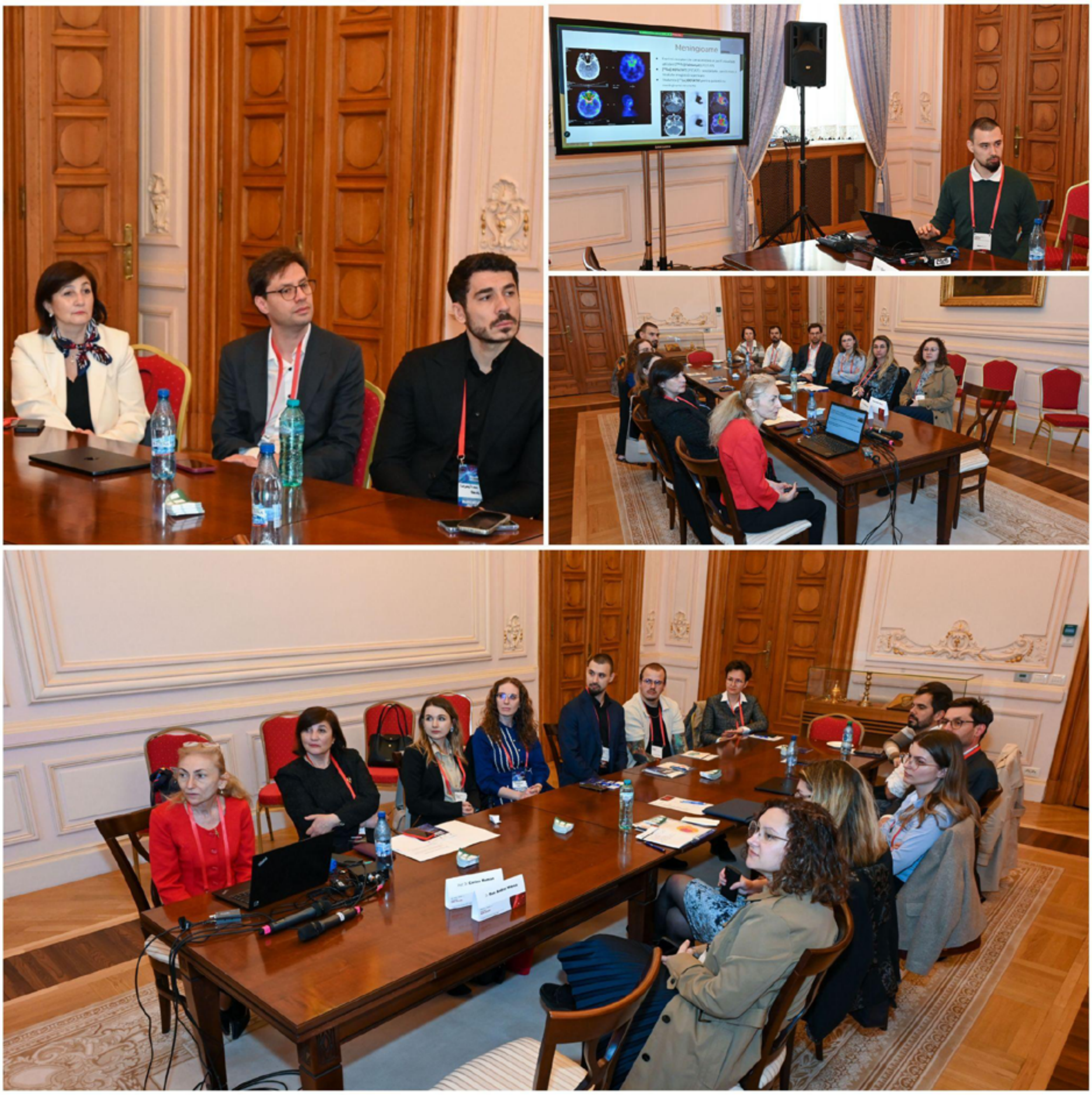

F. Photos from the Neuro-oncology Workshop, coordinated by Prof. Dr Corina Roman, professor at the Department of Clinical Medicine, Faculty of Medicine, Lucian Blaga University in Sibiu & Dr Dan Mitrea, neurologist at Pierre Wertheimer Neurological Hospital in Lyon

© 2026 by the authors. This article is an open access article distributed under the terms and conditions of the Creative Commons Attribution (CC BY 4.0) license.

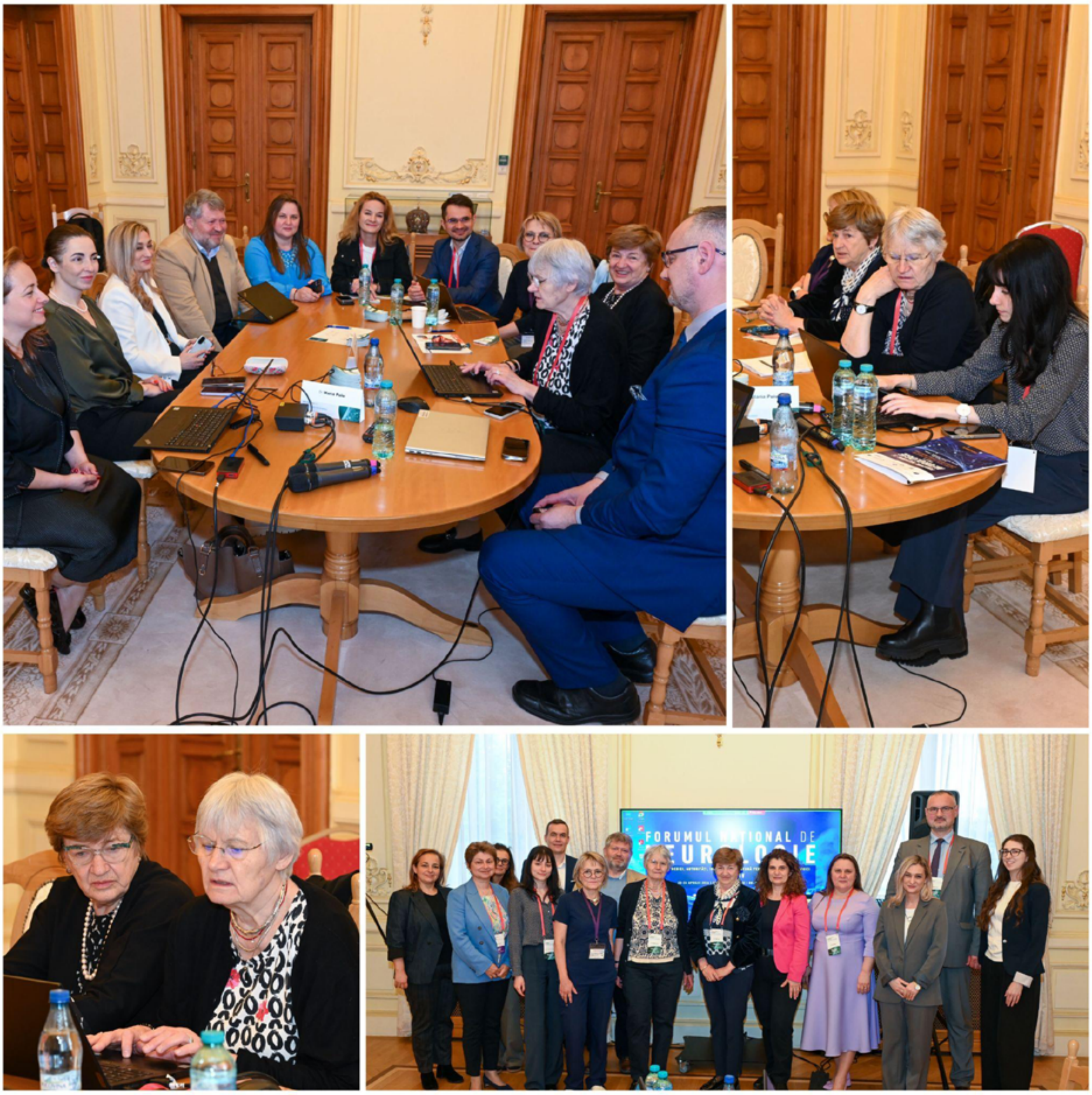

G. Photos from the Rare Neurological Diseases in Romania Workshop, coordinated by Prof. Dr Mihaela Simu, Senior Consultant Neurologist at NeuroTim Med & Prof. Dr Maria Puiu, Senior Consultant in Medical Genetics, Founding Member of the National Alliance for Rare Diseases Romania, and Coordinator of the Center for Genomic Medicine

© 2026 by the authors. This article is an open access article distributed under the terms and conditions of the Creative Commons Attribution (CC BY 4.0) license.

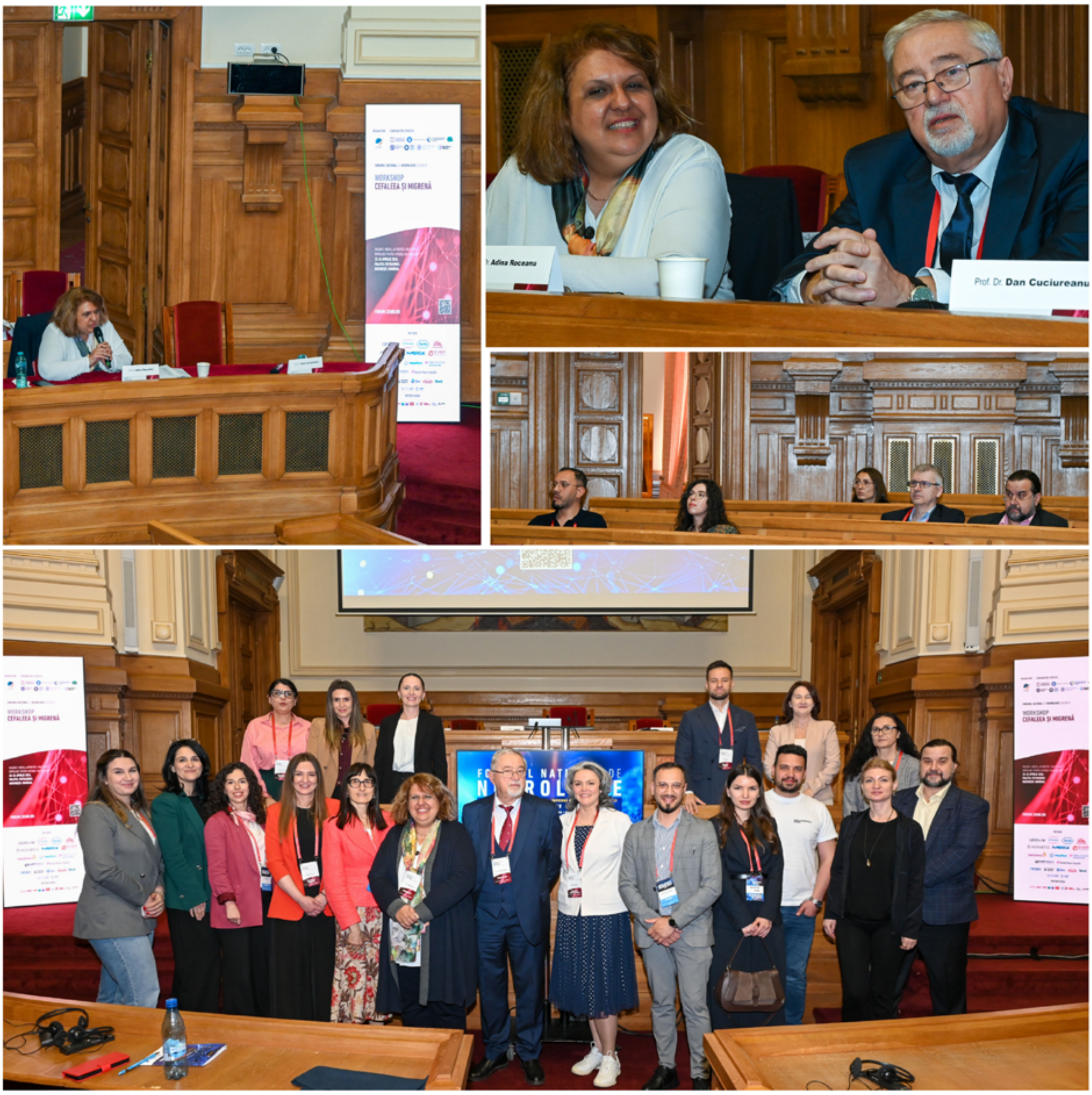

H. Photos from the Headache and Migraine Workshop, coordinated by Prof. Dr Dan Cuciureanu, neurologist at Neurology Clinic I from Emergency Clinical Hospital Prof. Dr. N. Oblu in Iași and the Head of the Department of Neurology at the Grigore T. Popa University of Medicine and Pharmacy of Iași & CSII Dr. Adina Roceanu, neurologist at the Department of Neurology from Bucharest University Emergency Hospital.

© 2026 by the authors. This article is an open access article distributed under the terms and conditions of the Creative Commons Attribution (CC BY 4.0) license.

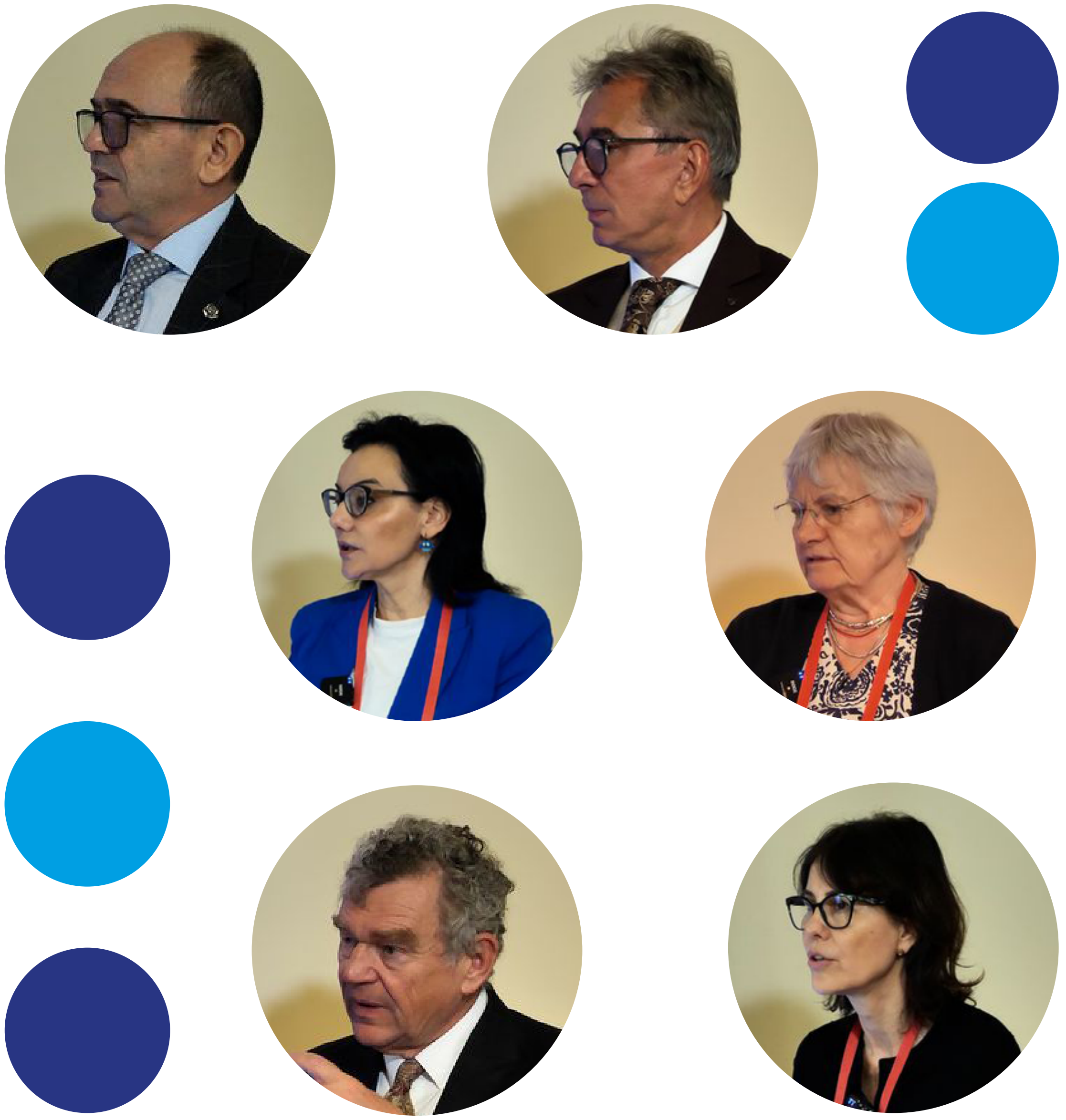

I. Photos from the Interviews during the 4th National Neurology Forum

© 2026 by the authors. This article is an open access article distributed under the terms and conditions of the Creative Commons Attribution (CC BY 4.0) license.

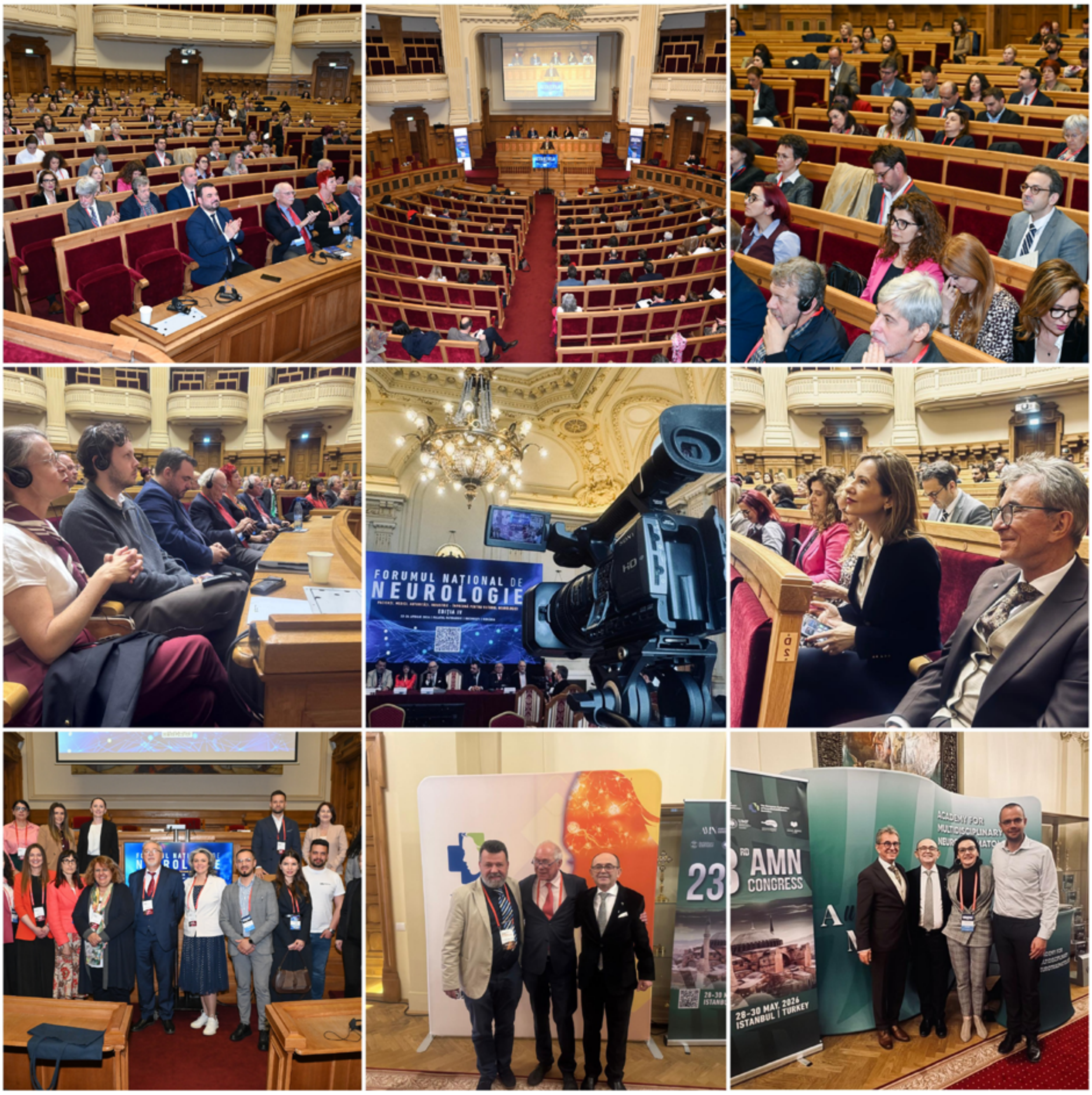

J. Photographs with participants and speakers at the National Neurology Forum

© 2026 by the authors. This article is an open access article distributed under the terms and conditions of the Creative Commons Attribution (CC BY 4.0) license.

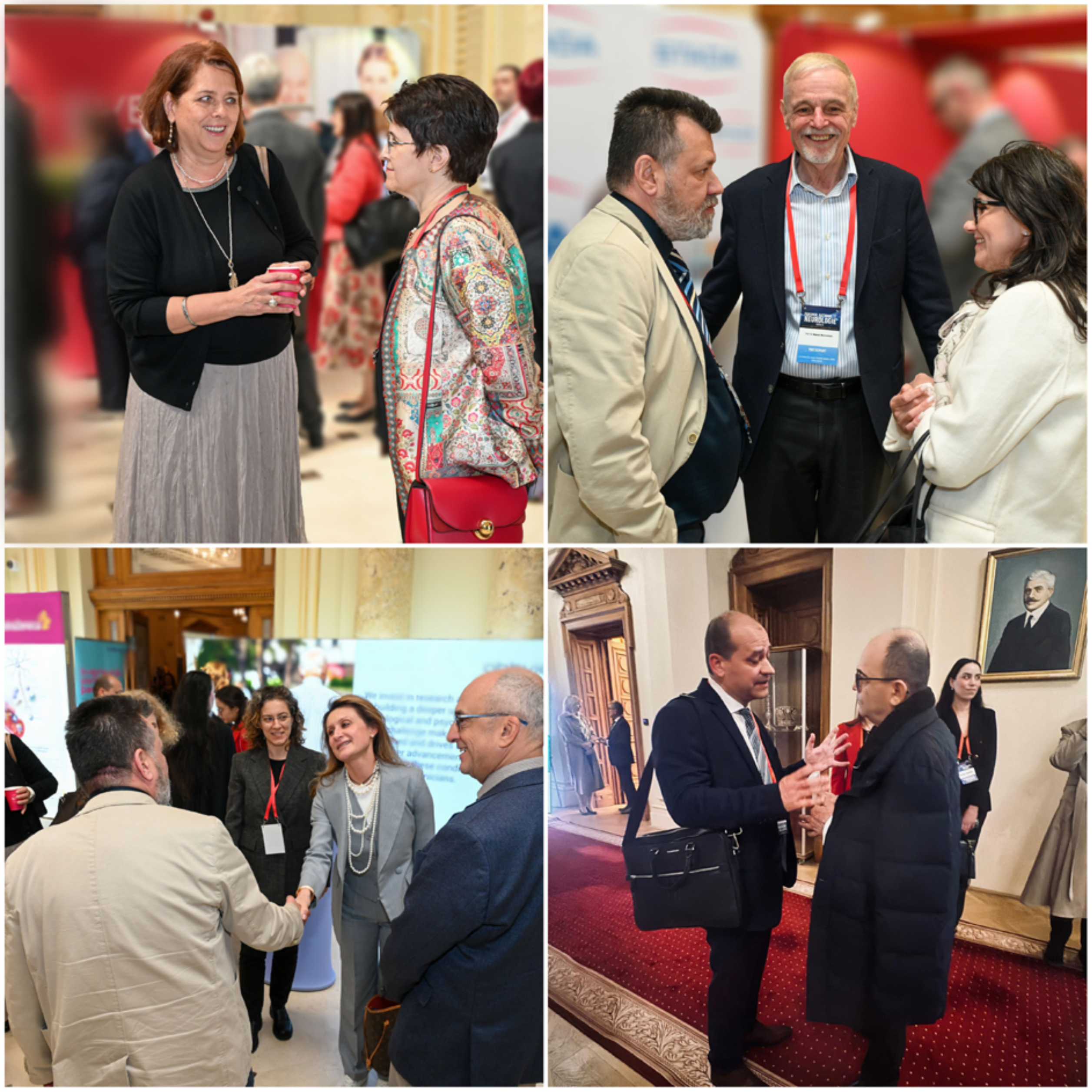

K. Photographs from the 4th National Neurology Forum

© 2026 by the authors. This article is an open access article distributed under the terms and conditions of the Creative Commons Attribution (CC BY 4.0) license.
